# The real-life costs of emotion regulation in anorexia nervosa: a combined ecological momentary assessment and fMRI study

**DOI:** 10.1038/s41398-017-0004-7

**Published:** 2018-01-24

**Authors:** Maria Seidel, Joseph A. King, Franziska Ritschel, Ilka Boehm, Daniel Geisler, Fabio Bernardoni, Larissa Holzapfel, Stefan Diestel, Kersten Diers, Alexander Strobel, Thomas Goschke, Henrik Walter, Veit Roessner, Stefan Ehrlich

**Affiliations:** 1 Division of Psychological and Social Medicine and Developmental Neuroscience, Faculty of Medicine, Technische Universität Dresden, Dresden, Germany; 2 Translational Developmental Neuroscience Section, Eating Disorder Research and Treatment Center, Department of Child and Adolescent Psychiatry, Faculty of Medicine, Technische Universität Dresden, Dresden, Germany; 3 International School of Management and Technical University of Dortmund, Dortmund, Germany; 4 Department of Psychology, Technische Universität Dresden, Dresden, Germany; 5 Department of Psychology, Charité Berlin, Berlin, Germany; 6 Department of Child and Adolescent Psychiatry, Faculty of Medicine, Technische Universität Dresden, Dresden, Germany

## Abstract

Regulation of emotions is necessary for successful attainment of short-term and long-term goals. However, over-regulation may also have its costs. In anorexia nervosa (AN), forgoing food intake despite emaciation and endocrine signals that promote eating is an example of “too much” self-control. Here we investigated whether voluntary emotion regulation in AN patients comes with associated disorder-relevant costs. Thirty-five patients with acute AN and thirty-five age-matched healthy controls (HCs) performed an established emotion regulation paradigm during functional magnetic resonance imaging after an overnight fast. The task required reducing emotions induced by positively valenced pictures via distancing. We calculated a neural regulation score from responses recorded in a reward-related brain region of interest (ventral striatum; VS) by subtracting activation measured on “positive distance” trials from that elicited under the “positive watch” (baseline) condition. Complementing the imaging data, we used ecological momentary assessment (EMA) to probe disorder-related rumination and affect six times/day for 2 weeks following the scanning session. The neural regulation score indicating reduced VS activation during emotion regulation was used as a predictor in hierarchical linear models with EMA measures as outcomes. No group differences in neural activity were found for the main contrasts of the task. However, regulation of VS activity was associated with increased body-related rumination and increased negative affect in AN, but not in HC. In line with this finding, correlational analysis with longitudinal BMI measurements revealed a link between greater VS regulation and poorer treatment outcome after 60 and 90 days. Together, these results identify a neural correlate of altered emotion regulation in AN, which seems to be detrimental to psychological well-being and may interfere with recovery.

## Introduction

Effective behavioral and emotional self-regulation is critical for the success of everyday functioning and health^1–4^. Research on self-regulation has mainly focused on failures of volitional control, such as in problem gambling, substance abuse, or obesity^5,6^. In contrast, clinical observations of patients with anorexia nervosa (AN) are suggestive of relatively elevated self-control. One of the most puzzling questions is how patients are able to abstain from food intake despite extreme low body weight and endocrine signals that promote eating^7,8^. In addition, primary rewards (food and sex) are often avoided by AN patients^9–12^. One hypothesis is that AN patients downregulate their responses to rewarding stimuli, which would be in line with reward-centered models of AN^13–15^ and a growing body of literature implicating alterations in activity and connectivity of reward-related mesolimbic brain structures such as the ventral striatum (VS) in AN^16–18^. Complementing these observations, some fMRI studies have found alterations in frontoparietal networks involved in cognitive control and executive functions^19–23^.

Researchers have proposed that not only a lack of, but also elevated self-control might come with associated costs^24,25^. This might also be the case for emotion regulation. Walter et al.^26^ demonstrated that initial successful downregulation of amygdala activity (measured using fMRI) via a reappraisal strategy (detachment; i.e., regulating emotion by adopting the perspective of a distanced and uninvolved observer)^27,28^ was followed by a paradoxical increase of amygdala activity during a rest period immediately after stimulus presentation. This finding underscores the notion that cognitive emotion regulation strategies might also have their costs. Regarding the regulation of positive emotions, distancing has been shown to be effective in downregulating reward-related feelings^29–31^, as well as in neural activation in the VS^30,32^. However, to date little is known about the costs of downregulating positive emotions.

Studies in healthy volunteers have emphasized the association between self-control, eating, and weight^33,34^. Indeed, clinical observations and data collected using ecological momentary assessment (EMA) suggest that, despite showing avoidance behaviors and regulating feelings of hunger, AN patients constantly ruminate about food and their bodies^35–37^. Also, research on restrained eaters or externally imposed food restriction indicates that chronic restriction of eating leads to emotional problems, increased distractibility, and obsessions with food^38–40^. Other studies also found an increase in eating disorder (ED) symptoms after using thought suppression as a regulation strategy^41–43^, suggesting possible consequences in ED samples.

To shed light on the possible costs of excessive volitional control of reward-related processes in AN, a sample of acute patients and age-matched healthy controls (HCs) were asked to regulate emotions elicited by viewing positive pictures (e.g., puppies, happy family scenes, or fun sport) during fMRI. The focus of the current study was to assess the possible associations between regulation success (as gauged by the level of reduction in activity of the VS) and both short-term (affective states and ruminations) and long-term (weight gain) outcome measures. This is an important open question because measuring current, momentary effect in patients in natural settings may provide a more accurate index of true affective experience than questionnaires administered in the laboratory^44^. We employed a modified version of an established emotion regulation paradigm^26,27,45–47^, which focuses on distancing as an emotion regulation strategy. We hypothesized that increased regulation of emotions elicited by rewarding stimuli (positive pictures) in patients would be associated with an increase in intrusive disease-relevant thoughts (food/body) as well as with negative effect and tension measured in the natural environment. Further, we expected higher regulation success to be associated with less favorable long-term outcome as measured in follow-up body mass index (BMI) in the patient group.

## Materials and methods

### Participants

The sample in the current study consisted of a total of 70 female volunteers: 35 patients with acute AN according to DSM-V (12.1–29.2 years old) and 35 HC (12.1–29.5 years old). AN patients were admitted to ED programs of a university child and adolescent psychiatry and psychosomatic medicine department and were assessed and scanned within 96 h after the beginning of a behaviorally oriented nutritional rehabilitation program. To be included in the HC group, participants had to be of normal weight and eumenorrhoeic. Normal weight was defined as BMI equal or above the 10th age percentile (if 18 years or younger)/BMI equal or above 18.5 kg/m^2^ (if older than 18 years), or below the 94th age percentile (if 18 years or younger)/BMI below 28 kg/m^2^ (if older than 18 years). HCs were recruited through advertisement among middle school, high school, and university students. Exclusion criteria and possible confounding variables for both groups were obtained using a semi-structured research interview (SIAB-EX), our own semi-structured interview, and medical records if applicable (for further details of exclusion and inclusion criteria of both groups, as well as partial overlap with our previous study Seidel et al.^36^ please see Supplementary Material 1.1).

Study data were collected and managed using secure, web-based electronic data capture tools REDCap (Research Electronic Data Capture)^48^. This study was approved by the local Institutional Review Board, and all participants (and if underage their guardians) gave written informed consent.

### Clinical measures

To complement the information obtained with the clinical interviews, we assessed ED-specific psychopathology using the Eating Disorder Inventory (EDI-2)^49^ and depressive symptoms using the Beck Depression Inventory^50^. For habitual use of the emotion regulation strategies' reappraisal and suppression, we used the Emotion Regulation Questionnaire (ERQ)^51^. BMI, gender, and age-corrected BMI-standard deviation score (BMI-SDS)^52^ development of patients was measured at the day of scanning and after 30, 60, and 90 days after admission to the inpatient rehabilitation program.

### Emotion regulation task

During the task (see Supplementary Fig. S1), participants were asked to either passively view the set of negative, positive, and neutral pictures or to actively downregulate any emotions arising in response to the negative and positive pictures. Negative, positive, and neutral stimuli for the emotion regulation task were selected from the International Affective Picture System^53^ and the emotional pictures set (Emopics)^54^. All stimuli were presented onto a back-projection screen located at the rear end of the scanner and were viewed through a mirror attached to the head coil. During the view condition, participants were instructed to simply view the picture without modulating any associated feelings, while not to look away or distract themselves in any way. During the regulation condition they were told to try to downregulate any elicited feeling via the reappraisal strategy “distancing”. More specifically, they were instructed to detach themselves from the upcoming emotion: “Look at the following picture directly, but try to take the position of a non-involved observer/thinking about the present picture in a neutral way/imagine that between you and the picture is a wall of glass/imagine the picture is getting smaller and smaller”.

The instruction for each condition was given by presenting a cue word laid over the stimulus for 1.5 s, stating either “view” or “distance”. We did not include a “distance neutral” condition in the experiment because of an assumed lack of validity of this condition; we expected no initial emotional reaction that allowed being downregulated. After each picture presentation, participants were asked to rate how aroused they were at the current moment on a visual analog scale, ranging from “very aroused” to “not aroused at all”.

To ensure understanding of the instructions and familiarity with the procedure, all participants underwent a training session outside the MR scanner, which took about 10 min and consisted of 17 trials, training each condition. After completion, they were asked whether they had any difficulties applying the instructions and to explain what they had done during the regulation instruction. If this report was incompatible with prior instructions or participants reported difficulties with the task, instructions were read again and participants were asked to do another training session. All stimuli used in training were different from those shown in the main experiment. The complete task consisted of 100 trials (20 per condition), which were presented in pseudorandomized order with each condition constrained to not occur more than twice in a row, while the assignment of stimuli to either the “view” or “distance” condition was randomized for each participant. The subsequent fMRI measurement lasted for ~23 min. In line with the hypotheses, the current study focused on the regulation of the positive stimuli.

### EMA

For more specific information regarding the EMA sampling procedure, please refer to Seidel et al.^36^ or to the Supplementary Material 1.3. In short, EMA sampling via smartphones started the day after the fMRI scan for a period of 14 days. Alarms occurred at six semi-random times during a 14-h period that was adapted for each individual to suit different daily routines.

Each prompt examined rumination about AN-related content (food/weight), which was assessed via two items adapted from the SIAB-EX interview (question 60 and question 61), i.e., “How much have you been thinking about food/calories/cooking?” and “How much have you been thinking about your weight/shape?”. Responses were given on a visual analog scale, ranging from “not at all” to “a lot”.

An adapted version of the Multidimensional Mood Questionnaire^55^ recommended to use in EMA research^56^ assessed tension, affect, and energetic arousal with two bipolar items each. Participants were asked to rate on a visual analog scale how they had felt since the last alarm. Higher scores indicated less negative affect, less tension, and higher energetic arousal.

### Functional image acquisition and processing

Images were acquired between 8 and 9 a.m. following an overnight fast using standard sequences with a 3 T whole-body MRI scanner (TRIO, Siemens) equipped with a standard head coil (see Supplementary Material 1.4). Functional and structural images were processed with SPM8 (www.fil.ion.ucl.ac.uk/spm) within the Nipype framework (http://nipy.sourceforge.net/nipype/37) following standard procedures, an artifact detection tool, and DARTEL (see Supplementary Material 1.4).

On the single participant level a general linear model was fit to model the brain activation in response to each of the five conditions (neutral, positive/negative watch, positive/negative distance). We modeled the picture as boxcar function with a duration of 6 s and the subsequent rating as stick-function (zero duration). Additional regressors included six motion parameters and one regressor for each motion or intensity outlier volume (for details see Supplementary Material 1.4) as nuisance regressors of no interest. All events were modeled using a canonical hemodynamic response function. At the second level, we conducted independent *t*-tests to assess group differences between the individual contrasts.

On the basis of the role of the VS in reward processing in the context of emotion regulation^30^ and our research question, whether there are AN-relevant consequences of regulating reward-associated emotions, the bilateral VS as defined by the AAL atlas and implemented in the WFU PickAtlas toolbox for SPM^57,58^ was used as a region of interest (ROI). To control for false-positives regarding main effects (including both groups) and group differences in the VS, family-wise error (FWE, *p* < 0.05) correction was performed using small volume correction in SPM8. Whole-brain analysis of main effects and group differences of interest (positive watch > neutral; positive watch > positive distance; positive watch < positive distance) were also corrected at a threshold of *p* < 0.05, FEW using random field theory^59^. We further averaged extracted indices of activation (β estimates) using the MarsBaR toolbox^60^ from the ROI VS.

## Statistical analysis

### Clinical and psychometric data

We conducted a 3 × 2 repeated measures ANOVA to test for potential group differences in the effect of the three conditions of interest (neutral, positive watch, and positive distance) on the arousal ratings. To investigate effects between reported regulation and imaging data, we calculated an arousal regulation score by subtracting the rated arousal following “positive distance” trials from those following “positive watch”. The higher this arousal regulation score, the more the arousal was reduced during the regulation condition as compared to the watch condition. To assess outcome measures, we calculated BMI-SDS change scores by subtracting BMI-SDS after 30, 60, and 90 days from BMI-SDS at the day of admission. Histograms, box plots, normal probability plots, and Levene statistics were employed to verify the underlying statistical assumptions—no major deviations were detected.

### fMRI

To investigate effects of neural regulation on the EMA data and outcome measures, we calculated a neural regulation score by subtracting the betas extracted from the VS ROI in the “positive distance” condition from those during “positive watch”. The higher this neural regulation score, the more neural activity was reduced during the regulation condition as compared to the watch condition. To assess whether this neural regulation score was associated with the arousal regulation score or clinical characteristics, Pearson’s correlation was calculated if data were normally distributed, otherwise Spearman’s rho was used. To investigate the relationship between VS neural regulation and weight gain after 30, 60, and 90 days, we conducted three linear regressions with the respective change in BMI-SDS as outcome variable and added the VS neural regulation score, baseline BMI-SDS, and baseline EDI-2-total as predictors. To reach normal distribution of the neural regulation score for inclusion in the hierarchical models, one outlier was removed from the data set.

### Hierarchical linear models

As the research design of the EMA data yields nested data, we conducted hierarchical linear modeling (HLM 7)^61^ to examine the extent to which the neural regulation score was able to predict disease-relevant ruminations, affect, and tension in the 14 days following the scan.

For all outcome variables (rumination about food, rumination about weight and shape, negative affect, and tension) separate multilevel models were estimated. These models take into account that the present data set is organized within three different levels and that single observations (Level 1) are nested within days (Level 2), which are nested within participants (Level 3). The same statistical approach was used for all models.

First, a null model including intercepts and error terms, but without predictors on any level, was calculated to analyze within-person variability. In model 1a, 1b, 1c, and 1d (for our four different outcomes) we allowed for random intercepts and included time (indicating time of day as a continuous variable from 1 to 6) on level 1 and day of study (1–14) on level 2. To account for autoregression, we estimated random slopes for the time variables at level 1 (time points per day) and level 2 (days)^62^. On level 3, the person level, we included diagnostic group (−1 and 1), mean centered extracted beta values of the VS ROI, as well as an interaction term of group and the centered VS neural activity. To facilitate the interpretation of the interaction patterns, we performed simple slope tests, as recommended by Preacher, Curran, and Bauer^63^. To explore whether group differences revealed in associations between VS regulation and EMA data represented a generic response in brain regions involved in emotion regulation and reward processing, we carried out control analyses of relationships between EMA variables and change in BOLD response in the amygdala^64,65^; a brain region tightly connected to VS and previously implicated in AN^66,67^ by adding a neural regulation score of the amygdala as predictor to the HLM models instead of the VS regulation score.

## Results

### Demographic and clinical variables, self-report data

AN patients did not differ from matched HC in age or IQ, but BMI was significantly lower and ED symptoms as well as depression scores were considerably elevated (Table 1). Further, as indicated by their scores on the subscales of the ERQ, patients were less likely to habitually use reappraisal than HC when regulating emotions, while no differences were observed for the suppression subscale (Table 1).

**Table 1:** Demographics: mean (SD)

	AN (*n* = 35)	HC (*n* = 35)
Age	16.48 (3.73)	16.73 (3.81)
Compliance	85.13 (12.13)	76.1 (14.25)**
BMI	14.65 (1.25)	20.67 (2.43)***
BMI-SDS	−3.24 (1.07)	−0.09 (0.71)***
Duration of illness	10.86 (15.82)	
IQ	113.78 (10.87)	113.14 (6.4)
EDI-2	219.27 (42.08)	142.14 (30.6)***
BDI-II	23.26 (10.73)	6.33 (5.43)***
ERQ-reinterpretation	24.15 (6.39)	27.43 (6.39)*
ERQ-suppression	14.44 (4.83)	13.6 (4.93)

Results of two-sided independent *t*-test for group comparison. Age is given in years, BMI in kg/m², compliance in %, duration of illness in months. *AN* anorexia nervosa patients. *HC* healthy controls, *BMI* body mass index, *BMI-SDS* body mass index standard deviation score, *IQ* intelligence quotient, *EDI-2* eating disorder inventory (total score), *BDI-II* Beck depression inventory, *ERQ* emotion regulation questionnaire. *p < 0.05, ***p < 0.001

**Fig. 1:**
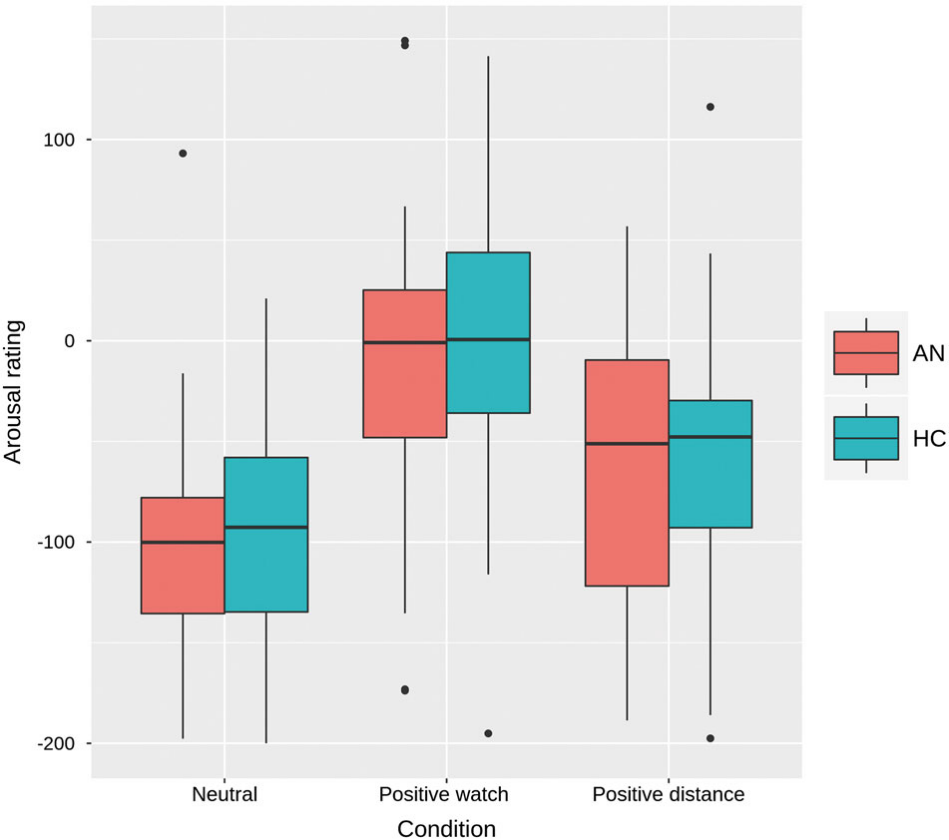
Subjective ratings. Arousal ratings displayed as box plot with median, first, and third quartile and the 95% confidence interval for each group during the emotion regulation task for the conditions neutral watch, positive watch, and positive distance. Rating scale ranges from −200 (not aroused) to +200 (very aroused)

Regarding the arousal ratings during the fMRI experiment, an expected main effect of condition was found (F(2,140) = 61.29, *p* < 0.001). Post hoc tests confirmed that arousal in the positive watch condition was higher than in the neutral watch condition (*p* < 0.001) and that it decreased during the emotion regulation condition compared to watch (*p* < 0.001), but no interaction with group was evident (see Fig. 1). The regulation score (positive watch–positive distance) as gauged by the arousal ratings did not show any correlations with age, BMI-SDS, EDI-2-total, or depression scores (Supplementary Table S1).

### Imaging data

Exploratory whole-brain analyses of the main effects of emotion and task, which were not in the focus of the current study, are reported in the Supplementary Fig. S2 and Supplementary Table S2). No group differences were evident on whole-brain level or in the VS ROI, neither during emotion induction (positive watch>neutral), nor during emotion regulation (positive watch>positive distance, positive watch<positive distance). Supporting the general validity of the paradigm, a positive correlation was evident between the extracted neural regulation score (VS, positive watch–positive distance) and the arousal regulation score (arousal ratings, positive watch–positive distance; rho = 0.27, *p* = 0.026; see also Supplementary Fig. S3). Examining the association between self-reported emotion regulation strategies (ERQ) and VS neural regulation score, we found a positive association between the neural regulation score and self-reported habitual use of suppression in patients only (AN: *r* = 0.49. *p* = 0.003; HC: *r* = −0.14, n.s.). No correlation between reappraisal and neural regulation score surfaced (AN: *r* = 0.25, n.s.; HC: *r* = −0.13, n.s.).

**Fig. 2:**
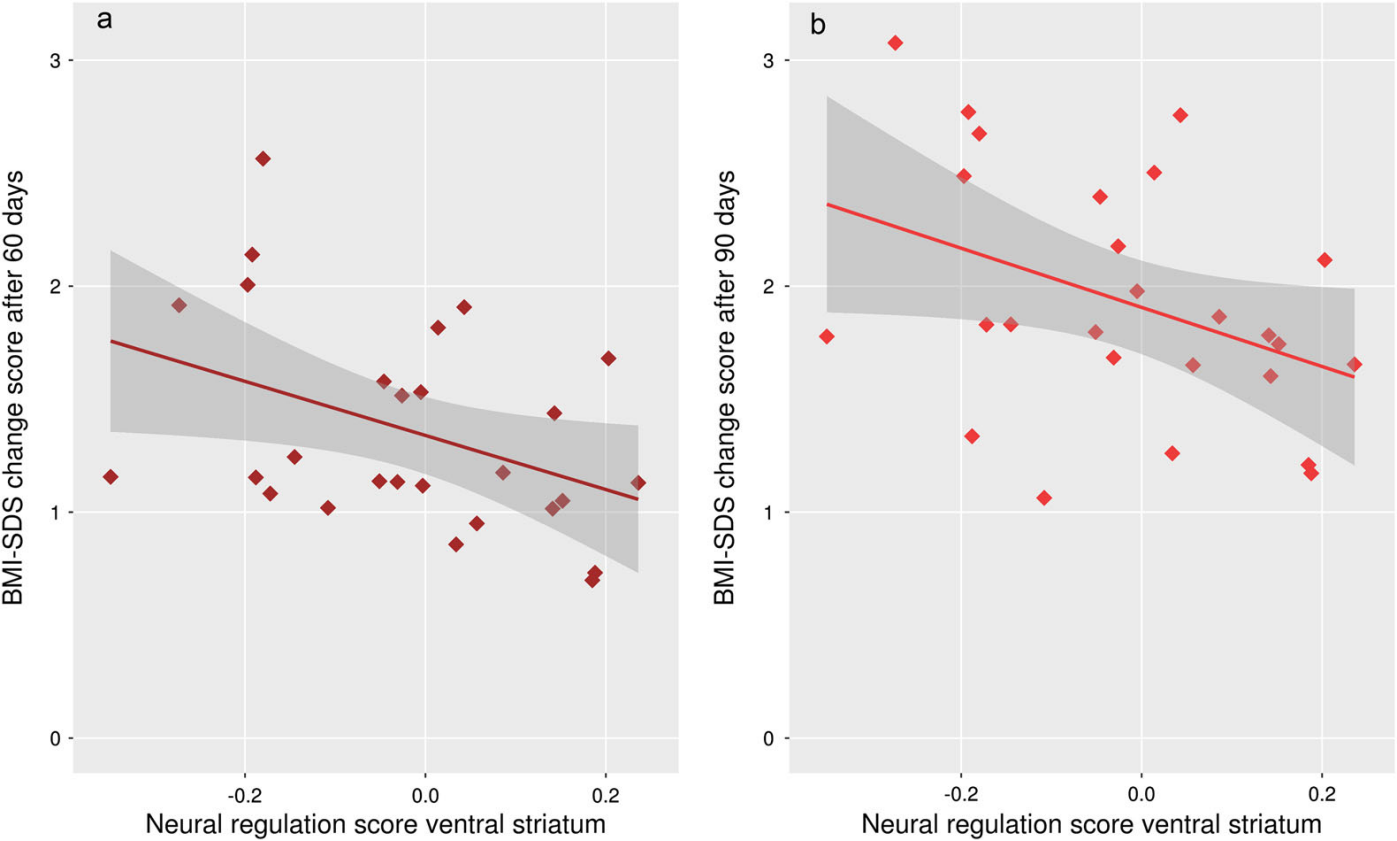
Association between BMI outcome and neural regulation. Correlation between BMI outcome after 60 days **a** and 90 days **b** and neural regulation score. Results were replicated using linear regression additionally accounting for EDI-2-total and BMI-SDS at baseline (see Supplementary Table S3a/S3b)

More importantly, VS neural regulation score predicted treatment outcome after 60 (*r* = −0.36, *p* = 0.031) and 90 days (*r* = −0.47, *p* = 0.006; Fig. 2 and Supplementary Table S3) in patients beyond the effects of baseline BMI-SDS and baseline EDI-2-total, indicating that the more patients reduced VS activity during the “positive distance” condition as compared to “positive watch”, the less weight they had gained after 60 and 90 days.

### Hierarchical linear models

We tested the effect of the neural regulation score on subsequent rumination about weight, rumination about food, affect, and tension with four different models, each was based on a total of 4594 (AN = 2434, HC = 2160) data points. The null model of all outcomes (intercept as the only predictor) showed significant variance at both higher levels, hence justifying the application of further multilevel analysis (Supplementary Table S4). In model 1, we controlled for time of day, day of study, and diagnostic group, for effects of neural regulation in the VS as well as the interaction term neural regulation score × group on the respective outcome measure (for additional models also controlling for age, compliance rates, duration of illness, and AN subtype, please refer to Supplementary Tables S5 and S6).

As expected, the diagnostic group showed a significant effect on the intercepts of rumination about weight, rumination about food, affect, and tension. Rumination as well as negative affect and tension were higher for AN compared to HC. Results further indicated that there was a significant main effect of neural regulation on rumination about weight, and most importantly, a significant neural regulation score × group interaction for rumination about weight, affect, and tension (for cross-level effects of time and day, see Table 2, for effect sizes see Table S7). These effects were not present for the arousal regulation score or the neural regulation score of the amygdala (see Supplementary Tables S8 and S9).

**Table 2:** Multilevel estimates for models predicting rumination (food, weight), affect, and tension

Parameter	Food			Weight			Affect			Tension		
	Beta	SE	*p*	Beta	SE	*p*	Beta	SE	*p*	Beta	SE	*p*
Fixed effects												
Intercept	44.15	2.45	<0.001	38.01	2.61	<0.001	117.36	4.13	<0.001	122.98	4.74	<0.001
Group	13.69	2.45	<0.001	21.08	2.61	<0.001	−31.62	4.13	<0.001	−18.65	4.74	<0.001
Day	0.14	0.16	n.s.	0.31	0.17	n.s.	0.1	0.3	n.s.	0.34	0.28	n.s.
Day × Group	0.21	0.16	n.s.	0.33	0.17	n.s.	−0.56	0.3	n.s.	−0.62	0.28	0.033
Time	0.58	0.23	0.015	0.13	0.2	n.s.	0.51	0.42	n.s.	0.21	0.41	n.s.
Time × Group	−0.58	0.23	0.016	−0.35	0.2	n.s.	0.00	0.42	n.s.	0.07	0.41	n.s.
Neural regulation	21.95	10.40	0.039	22.99	9.77	0.022	−5.06	14.34	n.s	8.38	15.45	n.s.
Neural regulation × group	15.62	10.40	n.s.	23.07	9.77	0.021	−46.64	14.34	0.002	−38.29	15.46	0.016
*Random effects*												
* σ*² = residual variance at Level 1	435.68			287.42			943.51			960.44		
* τ*² intercept = residual variance at level 2	16.49			31.69			258.25			206.55		
* μ*² intercept = residual variance at level 3	367.68			436.95			1014.9			1376.49		
*Model comparison*												
−2×log (lh)	42,053.48***			40,404.43***			46,260.65***			46,257.97***		
Diff−2×log (lh)	155.51			237.51			187.64			128.56		
Number of parameters	18			18			18			18		

*n*. *SE* standard error, *Group* −1(HC) 1(AN), *Day* day within study, *Time* prompt within day, *Neural regulation score* positive watch−positive distance of extracted parameter estimates of ventral striatum. *p < 0.05, **p < 0.01, ***p < 0.001

**Fig. 3:**
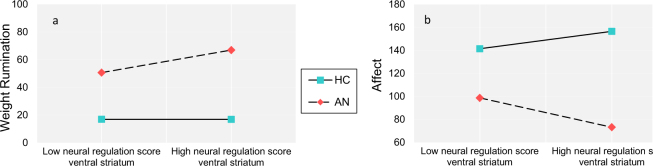
Modelled association between neural regulation, rumination and affect. **a** Neural regulation score × group interaction effect for rumination about weight as modeled by HLM. Simple slope analysis: HC: *t* = 0, *p* = 0.99, AN: *t* = 2.96, *p* = 0.004. Low values indicate low rumination. **b** Neural regulation × group interaction effect for affect as modeled by HLM. Simple slope analysis: HC: *t* = 2.38, *p* = 0.018, AN: *t* = 2.12, *p* = 0.034. Low values indicate more negative affect. *AN* anorexia nervosa; *HC* healthy control; Neural regulation score of ventral striatum is dichotomous for display purposes only

Slope analysis revealed that the VS neural regulation score predicted rumination about weight in the AN sample only (Fig. 3a): the higher the regulation score, the higher the subsequent rumination measured over 2 weeks post scanning (*p* = 0.004). The interaction between neural regulation and diagnostic group for affect was driven by a larger neural regulation score being associated with higher negative affect in the AN sample (*p* = 0.034; Fig. 3b) and a larger regulation score being associated with higher positive affect (*p* = 0.018; Fig. 3b) and lower tension (*p* = 0.015; Fig. S4) in HC over 2 weeks post scanning.

## Discussion

The goal of this combined fMRI/EMA study was to investigate whether regulation of positive emotions as gauged by the reduction of hemodynamic activity in a reward-related brain region (VS) was associated with negative consequences for AN patients in their everyday lives as measured by EMA. Indeed, the imaging data were predictive of patients’ momentary ratings of affect and rumination. Specifically, we found the neural correlates of downregulation of positive emotions in the VS to be closely related with real-life measures of disorder-relevant rumination as well as negative affect and tension scores measured several times a day over 2 weeks after the fMRI scan. Furthermore, the more VS activity was downregulated during distancing from positive emotions, the less weight AN patients gained until follow-up time points at 60 and 90 days into therapy. As discussed below in light of the ironic processes theory^68^ and accounts of limited neural control capacities^69^, we propose disorder-relevant negative outcomes to be the result of a maladaptive self-control mechanism closely related to AN symptomatology.

In the framework of their ironic processes theory, Wenzlaff and Wegner^70^ have conducted various studies showing that frequently suppressing thoughts or emotions can be maladaptive and ultimately ineffective as it often results in a paradoxical increase or rebound of the unwanted thoughts^68,71–74^. This rebound is often characterized by an increased accessibility of the suppressed thought^72^ and heightened emotional arousal^75^. It has also been suggested that chronic suppression might prevent habituation to emotional stimuli, and as such results in hypersensitivity to depression and anxiety-related thoughts and symptoms^70,73^. This is in line with previous findings of increased rumination in AN patients^35–37^ and might also explain the association between neural correlates of emotion regulation (as measured in the VS in the current study) and increased post-scan rumination about weight-related and body-related content.

Although the detachment strategy applied in our task belongs to the group of reappraisal strategies^28^, it seems possible that detachment and suppression share some common mechanisms^26,76^ or are used interchangeably by some patients. Indeed, the instructions of emotion regulation experiments in some studies reflect these similarities, with some stating to induce “suppression” of emotions via detachment^77^, while others instruct participants to “distance” themselves from upcoming emotions during a condition that is labeled “suppression”^78^. Others have also highlighted the importance of distinguishing between the reappraisal strategies of reappraisal as reinterpretation and reappraisal as detachment/distancing, with the latter being conceptually more similar to thought suppression^76,78–80^. The assumption that especially patients might tend to use both strategies interchangeably is also supported by the found association between the neural regulation score (acquired during “distancing”) and the suppression subscale of the ERQ in our AN group only. A possible interpretation of this finding might be that patients, who appear to have less access to adaptive strategies like acceptance, problem-solving or strategies involving reappraisal, might use suppression as an alternative in order to cope with their emotions. Studies have shown that reappraisal, in contrast to suppression, is not associated with negative consequences like rebound effects such as increased ruminative thinking and more negative affect^81^. This rather speculative interpretation is supported by our finding in HC, who seemingly executed a reappraisal strategy (as requested) and therefore benefit from emotion regulation. In this group, a higher neural regulation score during the instructed reappraisal was associated with more positive affect and less tension post scan.

Affect plays a major role in eating-related behaviors^82^ and previous studies have found links between the reliance on suppression as an emotion regulation strategy and ED symptoms. Emotion suppression seems not only to result in increased rumination about food and eating-related content, but also be related to binge eating and food cravings among restrained eaters and individuals high in both restraint and disinhibition^41,43,71,83^. In clinical populations, it has been reported that thought suppression was associated with bulimic symptoms^42,84^. Moreover, studies and clinical observations suggest that AN patients are characterized by a general mode of avoidance toward intense emotional states^35,85–87^, even positive ones, which may also require constant monitoring and suppression of upcoming emotions. This is in line with evidence that the avoidance of various psychological experiences (thoughts, emotions, memories, or urges) are associated with ED symptoms such as binge eating and general psychological symptoms cross-sectionally and at follow-up^35,85,88–90^. Further, emotional avoidance in AN has been closely tied to negative affect and ruminative processes^35,86^. Considering the constant downregulation of hunger and food intake, as well as emotional avoidance typically found in AN^36,37,86^, a possible interpretation might be that these regulation strategies “backfire” by making the “over-controlled” stimuli even more accessible, which is reflected in heightened rumination, negative affect, and increased tension in AN as found with our EMA measures.

A similar interpretation of our findings comes from theories of self-control failure and “ego depletion”^69,91,92^. These theories have emphasized the costs and negative consequences of self-control within a limited capacity model^69^. Several experimental studies have yielded empirical evidence supporting this model by showing that performance on other cognitive tasks is worse after an emotion regulation condition, or that emotion regulation ability decreases with cognitive load^93,94^. This converges with results from studies in cohorts characterized by high dietary restraint. Participants of these studies have been shown to ruminate, crave, and overeat when asked to regulate emotions while cognitive resources are depleted^84,95,96^. Consequently, it has been suggested that emotion regulation depletes control resources^96^, which facilitates the emergence of ED-associated symptoms such as ruminative thinking or negative affect, which, as we were able to show, might even influence treatment outcome. The current findings, suggestive of over-regulation over reward-related processing in AN, are generally in line with recent findings of altered neural correlates of self-control in the disorder^97^ and may also be related to abnormal processing of positive socioemotional stimuli^98,99^. Although the current study did not explore potential evidence in the respect that the cortical regions typically involved in distancing (temporal parietal junction, inferior parietal lobule, and inferior frontal gyrus)^30,31^, directly modulated VS activity in AN, recent investigations of effective connectivity have shown altered top–down control within cognitive-emotional frontostriatal circuitry in AN^16,100^.”

The current study has to be considered in the light of a number of limitations: first of all, in order to avoid anxious mood states, we only investigated emotion regulation to a strictly filtered stimulus set. Emotions that are elicited through food pictures could be associated with even more regulation effort and therefore costs. Our analysis also does not address the association between regulation of negative stimuli and AN symptomatology. Second, although inter-individual differences in VS regulation during emotion regulation predicted disorder-relevant measures such as rumination, affect, and BMI outcome, when looking at the sample as a whole, we did not observe robust main effects of emotion and regulation. However, perceived emotion induction and regulation was successful for both groups. Third, the current results do not answer the question whether alterations in reward-associated brain areas represent a state-like response during the acute state of AN or a stable, trait-like difference in reward processing, and whether it changes with recovery and during therapy. Fourth, scanning after an overnight fast may have differential effects on AN patients, and HC thus may have modulated VS activation. Fifth, although we used a pairwise matching procedure regarding age, given the relatively large age range of the participants, age might have biased our results. Additional analyses with age as a covariate confirmed our initial results, but other possible confounders such as pubertal status or hormonal changes need to be taken into account by future studies and when interpreting the current findings. Last but not least, the majority of patients were treated as inpatients, which might have biased the naturalistic elements of data collection, mainly by preventing them to use restrictive eating as an emotion regulation strategy^101,102^.

The current investigation constitutes a first attempt to link underlying neural processes with real-life measures in AN. Because behavior and affect in laboratory studies may have little relevance to everyday life, establishing links between brain activity during controlled tasks and disorder-relevant cognition and affective states in natural settings is important when trying to elucidate the neural underpinnings of AN. Taken together, we established associations between neural responses of a reward-associated brain region during an emotion regulation task and rumination and negative affect measured in real life. Most therapeutic strategies deal with the acceptance of negative affect^103^. In contrast, our results seem to indicate the importance of a balanced way to deal with positive emotions. This could, for example, include the reintroduction of pleasurable feelings and joy during therapeutic approaches such as a meditation-based loving kindness intervention as part of the “Radically open-dialectical behavior therapy”, which has been recommended for AN^104^ as well as disorders of over-control^105^. Our results are compatible with the view that AN patients constantly downregulate, maybe suppress, pleasurable emotions, which seems to be associated with adverse therapy outcome. Allowing such responses (even if in response to food) or, if appropriate, using healthier emotion regulation strategies, could be an element that needs to be integrated into modern psychotherapeutic approaches to AN.

Although our findings need to be replicated, they seem to suggest that focusing on more adaptive emotion regulation strategies that rely less on control aspects that deplete cognitive resources, such as emotional acceptance^106^, may foster better overall psychological well-being and outcome success in patients with AN.

## Electronic supplementary material

Supplementary material
