# Supplementary material for: The real-life costs of emotion regulation in anorexia nervosa: a combined ecological momentary assessment and fMRI study
Source: Transl Psychiatry. 2018 Jan 24;8:28. doi: 10.1038/s41398-017-0004-7 (PMC5802555; doi:10.1038/s41398-017-0004-7)
Supplement: Supplementary file 1 — Supplementary material [file 41398_2017_4_MOESM1_ESM.doc]

**Seidel et al., The real-life costs of emotion regulation in anorexia nervosa: a combined ecological momentary assessment and fMRI study**

**Supplementary Material:**

1. Method

*1.1 Participants*

The diagnostic procedures, inclusion and exclusion criteria of the current study were identical to those in our previous behavioral study of rumination and affect in patients with anorexia nervosa (AN) ^1^. Information pertinent to inclusion and exclusion criteria (including possible confounding variables such as menstrual cycle and use of contraceptive medication) was obtained from all participants using the SIAB-EX semi-structured interview ^2^ conducted by clinically experienced and trained research assistants under the supervision of the attending child and adolescent psychiatrist. The 87 item interview systematically assesses the prevalence and severity of specific eating-related psychopathology and provides diagnoses according to ICD-10 and DSM-IV. This information was supplemented by our in-house semi-structured interview. Out of the 35 AN participants, three patients (8.6%) were of the binge-purge subtype. The rest of the sample was of the restrictive subtype. During the inpatient treatment, patients were enrolled in a comprehensive multimodal psychiatric and psychotherapeutic treatment program that consisted of individual, group, as well as family therapy. Central elements of the treatment were an operant behavioral program to achieve weight gain (>700g per week), cognitive-behavioral treatment, body-oriented psychotherapy, as well as relapse prevention. One of the included patients was started on a psychotropic medication (Zyprexa) during the EMA data collection and continued medication intake in the time period until the outcome assessments (weight measurements).

HC were not recruited if they had any history of psychiatric illness, a lifetime BMI below the 10^th^ age percentile (if younger than 18 years)/BMI below 18.5 kg/m^2^ (if older than 18 years), or were currently obese (above the 94^th^ age percentile (if 18 years or younger)/BMI above 28 kg/m^2^ (if older than 18 years)). Participants of both groups were excluded if they had a history of any of the following diagnoses: organic brain syndrome, schizophrenia, substance dependence, psychosis NOS, bipolar disorder, bulimia nervosa or binge-eating disorder. Further exclusion criteria for all participants were intelligence quotient IQ<85, psychotropic medication within six weeks prior to the study, current substance abuse, inflammatory, neurologic or metabolic illness, chronic medical or neurological illness that could affect appetite, eating behavior, or body weight, clinically relevant anemia, pregnancy or breast feeding.

Comorbid psychiatric diagnoses were made in AN patients by an expert clinician leading the treatment team and included examinations of the patients, discussions with the treatment staff and careful chart review (including consideration of medical and psychiatric history, SIAB-EX, physical examination, routine blood tests, urine analysis and several psychiatric screening instruments; see methods section in the main article). Four patients with AN reported having been in psychotherapeutic treatment for other psychiatric disorders than the eating disorder. One (2.8%) reported having had a depressive episode, three (3.8%) anxiety disorder, one (2.8%) obsessive compulsive disorder.

Because our nested data set requires multilevel modeling, we a priori defined a total sample size of at least 50 individuals. According to Hox and Maas ^3^ the sample size at the highest level (Level 3) should be higher than 50 units. Otherwise, biases in parameter estimation are likely. Additionally, there was an overlap between the current sample and that of Seidel et al., ^1^. Out of the 35 participants, four AN (11.4%) and out of 35 HC 12 (34.3%) were new and not included in our previous study.

- 1. *Clinical Measures*

We used a short version of the German adaptation of the Wechsler Intelligence Scale for Children ^4^, which included the following subtests: vocabulary, letter number sequencing, matrix reasoning, and symbol search. The short version of the German adaptation of the Wechsler Adult Intelligence Scale ^5^ included the subtests: picture completion, digit symbol coding, similarities and arithmetics.

To complement the information obtained with the clinical interviews, eating disorder-specific psychopathology was assessed with the German version of the Eating Disorders Inventory (EDI-2; (EDI-2) ^6^; here, we focused on the EDI-2 total score. Depressive symptoms were explored using the German version of the Beck Depression Inventory (BDI-II) ^7^.

*1.3 Ecological Momentary Assessment*

Participants had the choice between a study smartphone (Samsung Galaxy Ace, 5831i) and their own mobile phone (only one HC decided to use their own mobile phone). The app-based questionnaire was designed via an online platform (XS.Movisens, Karlsruhe, Germany), which also managed data collection and immediate server upload for constant monitoring of compliance.

Participants were initially screened, weighed, and interviewed, before they received detailed instruction on how to handle the smartphone, the Movisens-app and the content of the questionnaire. They were instructed to answer the questionnaire as soon as the alarm appeared, but were given an additional 30 minutes after the prompt when unable to reply (e.g., during class or work, during meal times or therapy session) or if safety was a concern (e.g., while driving).

EMA sampling started the day after the fMRI scan for a period of 14 days. Recordings during the first days were closely monitored in terms of compliance, to ensure that instructions were understood. Compensation was provided at the end of the study, in accordance with compliance rates.

Data collection was accomplished via the signal-contingent assessment method: Alarms occurred at six semi-random times during a 14 hour period that was adapted for each individual to suit different daily routines. Prompts were anchored within six smaller intervals of two 2 ½ hour intervals (before midday) and for 1 ½ hour intervals (after midday). Between the intervals were 30 minutes breaks, to ensure that three of the six meals provided during inpatient therapy were not included in the sampling.

*1.4 Imaging Acquisition*

Images were acquired with a Siemens 3T MRI scanner (Erlangen, Germany) equipped with a standard head coil. High-resolution structural images were acquired with a T1-weighted MPRAGE sequence (TR=1900 ms, TE=2.26 ms, FOV=256×256 mm, 176 slices, 1×1×1 mm^3^ voxel size, flip angle=9°). For functional imaging, a standard gradient-echo T_2_^*^-weighted EPI sequence was used (TR=2410 ms; TE=25 ms; flip angle=80°). A total of 575 volumes were obtained (42 transversal slices orientated 17° clockwise to the AC-PC line, 2 mm slice thickness, 1 mm gap, FOV=192×192 mm, in-plane resolution of 64×64 pixels=voxel size of 3×3×2 mm^3^). Task presentation and behavioral response recording was performed using Presentation software (Neurobehavioral Systems, Inc., Albany, CA).

Functional and structural data were processed using SPM8 (www.fil.ion.ucl.ac.uk/spm) within the Nipype framework (<http://nipy.sourceforge.net/nipype/>). Functional images were slice time corrected, realigned and registered to their mean. The preprocessed images were coregistered to the participant’s structural brain image. A DARTEL template ^8^ was created using the structural images from all participants. The functional volumes were normalized to Montreal Neurological Institute (MNI) space using the group template. The resulting data were smoothed with an isotropic Gaussian kernel (8mm FWHM). Prior to statistical analysis, we evaluated data quality by manual inspection and using artifact detection tools (ART; www.nitrc.org/projects/artifact_detect/) to identify volumes with intensity outliers (>3 SD from the mean of the time series) and excessive movement (>2 mm in any direction).

**Supplementary Tables and Figures**

**Table S1**

| Correlation coefficients of arousal regulation score with demographic and clinical variables | | |
| --- | --- | --- |
|  | **AN** | **HC** |
| **Age** | .05 | -.14 |
| **BMI** | .19 | -.18 |
| **EDI-2-total** | .01 | -.29 |
| **BDI-ll** | .01 | -.18 |

**Table S1:** AN=Acute anorexia patients, HC=Healthy controls, BMI=Body-mass-index, BMI-SDS=Body-mass-index standard deviation score, EDI-2-total=Eating Disorder Inventory (total score), BDI-II=Beck Depression Inventory. None of the correlations was significant at al alpha-level of .05.

Table S2

| Whole brain results |  |  |  |  |  |  |
| --- | --- | --- | --- | --- | --- | --- |
|  | cluster |  | cluster |  |  |  |
| **Label** | **p(FWE-corr)** | **k** | **p(unc)** | **x** | **y** | **z** |
| Positive watch > neutral |  |  |  |  |  |  |
| Inferior Occipital Gyrus | .000 | 3800 | .000 | 44 | -78 | -8 |
|  |  |  |  | 44 | -46 | -20 |
|  |  |  |  | 32 | -96 | 4 |
| Inferior Occipital Gyrus | .000 | 2530 | .000 | -38 | -84 | -8 |
|  |  |  |  | -40 | -48 | -16 |
|  |  |  |  | -42 | -58 | -16 |
| Middle Frontal Gyrus | .000 | 689 | .000 | -34 | 50 | 0 |
|  |  |  |  | -46 | 42 | 12 |
|  |  |  |  | -46 | 40 | 2 |
| Inferior Parietal Lobule | .002 | 533 | .000 | -62 | -36 | 44 |
|  |  |  |  | -52 | -40 | 54 |
|  |  |  |  | -44 | -54 | 58 |
| Amygdala/Hippocampus | .087 | 214 | .012 | 20 | -6 | -12 |
|  |  |  |  | 36 | 0 | -36 |
|  |  |  |  | 28 | 0 | -22 |
| Precuneus | .029 | 300 | .004 | 2 | -50 | 30 |
|  |  |  |  |  | -62 | 34 |
| Parahippocampus | .319 | 119 | .050 | -30 | -4 | -32 |
|  |  |  |  | -26 | -2 | -24 |
| Positive watch > positive distance |  |  |  |  |  |  |
| Cuneus/Calcarine/Middle Occipital Gyrus/Lingual Gyrus | .000 | 5047 | .000 | -20 | -98 | 16 |
|  |  |  |  | 14 | -98 | -6 |
|  |  |  |  | -2 | -102 | 10 |
| Medial Frontal Gyrus | .006 | 530 | .006 | 10 | 52 | 4 |
|  |  |  |  | -4 | 42 | -6 |
|  |  |  |  | -10 | 60 | 6 |
| Positive watch < positive distance |  |  |  |  |  |  |
| Supramarginal Gyrus | .00 | 841 | .00 | 62 | -52 | 34 |
|  |  |  |  | 54 | -50 | 54 |
| Inferior Frontal Gyrus | .03 | 370 | .00 | -54 | 24 | 2 |
| Middle Temporal Gyrus | .01 | 546 | .00 | -54 | -32 | -2 |
|  |  |  |  | -68 | -38 | 0 |
| Angular Gyrus | .01 | 529 | .00 | -56 | -56 | 30 |
|  |  |  |  | -42 | -54 | 24 |
| Superior Frontal Gyrus | .07 | 267 | .01 | 20 | 54 | 26 |
|  |  |  |  | 14 | 48 | 34 |
| Superior Frontal Gyrus/Supp Motor Area | .07 | 276 | .01 | 16 | 4 | 66 |
|  |  |  |  | 22 | -2 | 70 |
|  |  |  |  | 18 | 20 | 56 |

**Table S2:** Whole brain results for the contrasts positive watch>neutral, positive watch>posititive distance, positive watch<positive distance at a threshold of p<0.001 uncorrected, coordinates in Montreal Neurological Institute (MNI) space [x,y,z]. Clusters with a k>50 are included in the table.

**Table S3a/S3b**

Outcome: BMI-SDS change after 60 days

| S3a | Undstandardized Coefficients | | Standardized Coefficients |  |  |
| --- | --- | --- | --- | --- | --- |
|  | B | Std. Error | Beta | t | p |
| **(Constant)** | 0.56 | 0.44 |  | 1.28 | 0.211 |
| **EDI-2-total baseline** | 0.00 | 0.00 | -0.01 | -0.03 | 0.974 |
| **BMI-SDS baseline** | -0.26 | 0.09 | -0.45 | -2.88 | 0.008 |
| **VS neural regulation** | -0.97 | 0.42 | -0.36 | -2.27 | 0.031 |

Outcome: BMI-SDS change after 90 days

| S3b | Unstandardized Coefficients | | Standardized Coefficients |  |  |
| --- | --- | --- | --- | --- | --- |
|  | B | Std. Error | Beta | t | p |
| **(Constant)** | 0.25 | 0.60 |  | 0.42 | 0.679 |
| **EDI-2-total baseline** | 0.00 | 0.00 | 0.15 | 0.99 | 0.331 |
| **BMI-SDS at baseline** | -0.37 | 0.11 | -0.52 | -3.43 | 0.002 |
| **VS neural regulation** | -1.58 | 0.52 | -0.47 | -3.04 | 0.006 |

**Table S3a; S3b:** Results of linear regression to predict BMI-SDS Change after (a) 60 and(b) 90 days

**Table S4**

| Variance components of HLM Null Models | | | | | | | |
| --- | --- | --- | --- | --- | --- | --- | --- |
|  | **Final Estimation of Level-1 and**  **Level-2 variance components** | | |  | **Final Estimation of Level-3 variance components** | | |
|  | SD | Variance | p |  | SD | Variance | p |
| **Food** | 6.42 | 41.34 | <0.001 |  | 21.51 | 462.8 | <0.001 |
| **Level-1** | 21 | 441.07 |  |  |  |  |  |
| **Weight** | 8 | 64.02 | <0.001 |  | 28.45 | 809.44 | <0.001 |
| **Level-1** | 17.05 | 290.84 |  |  |  |  |  |
| **Affect** | 18.31 | 335.6 | <0.001 |  | 43.82 | 1919.77 | <0.001 |
| **Level-1** | 31.12 | 968.26 |  |  |  |  |  |
| **Tension** | 16.75 | 280.61 | <0.001 |  | 39.14 | 1531.8 | <0.001 |
| **Level-1** | 31.30 | 979.77 |  |  |  |  |  |

**Table S4:** Variance components of HLM null models for the outcomes rumination about food and weight, affect, and tension. SD=Standard deviation

| Multilevel estimates for models predicting rumination (food, weight), affect, and tension | | | | | | | | | | | | | | | | | | | |
| --- | --- | --- | --- | --- | --- | --- | --- | --- | --- | --- | --- | --- | --- | --- | --- | --- | --- | --- | --- |
|  | Food (a) | |  | | | Weight (b) | | |  | Affect (c) | | | |  | Tension (d) | | | | |
| Parameter | Beta | SE | | | p | Beta | SE | | p | Beta | SE | | p | | | Beta | SE | | p |
| **Fixed effects** |  |  | | |  |  |  | |  |  |  | |  | | |  |  | |  |
| Intercept | 37.69 | 16.98 | | | 0.030 | 38.00 | 14.57 | | .011 | 127.13 | 21.36 | | <.001 | | | 129.57 | 22.36 | | <.001 |
| Group | 13.81 | 2.67 | | | <.001 | 19.97 | 3.09 | | <.001 | -28.76 | 4.81 | | <.001 | | | -16.28 | 5.15 | | .002 |
| Age | 0.24 | 0.56 | | | n.s. | -0.58 | 0.42 | | n.s. | 0.48 | 0.62 | | n.s. | | | 0.17 | 0.78 | | n.s. |
| Compliance | 0.03 | 0.15 | | | n.s. | 0.12 | 0.15 | | n.s. | -0.20 | 0.23 | | n.s. | | | -0.09 | 0.26 | | n.s. |
| Duration of IIlness | -.045 | 0.15 | | | n.s. | 0.10 | 0.15 | | n.s. | -0.39 | 0.27 | | n.s. | | | -0.40 | 0.28 | | n.s. |
| Day | 0.14 | 0.16 | | | n.s. | 0.30 | 0.17 | | n.s. | 0.09 | 0.30 | | n.s. | | | 0.35 | 0.28 | | n.s. |
| Day×Group | 0.21 | 0.16 | | | n.s. | 0.33 | 0.17 | | n.s. | -0.56 | 0.3 | | n.s. | | | -0.62 | 0.28 | | .032 |
| Time | 0.58 | 0.23 | | | .015 | 0.13 | 0.20 | | n.s. | 0.51 | 0.42 | | n.s. | | | 0.21 | 0.41 | | n.s. |
| Time×Group | -0.58 | 0.23 | | | .016 | -0.35 | 0.20 | | n.s. | 0.00 | 0.42 | | n.s. | | | 0.07 | 0.41 | | n.s. |
| Neural Regulation | 20.78 | 12.42 | | | n.s. | 25.52 | 11.91 | | .036 | -15.30 | 18.29 | | n.s | | | -1.85 | 19.21 | | n.s. |
| Neural Regulation×Group | 14.47 | 12.16 | | | n.s. | 24.70 | 11.77 | | .040 | -55.72 | 18,95 | | .003 | | | -48.08 | 19.11 | | .014 |
| **Random Effects** |  | | |  |  |  | |  |  |  | |  |  | | |  | |  |  |
| σ² = Residual variance at Level 1 | 435.70 | | |  |  | 287.44 | |  |  | 943.54 | |  |  | | | 960.45 | |  |  |
| τ² Intercept = Residual variance at Level 2 | 16.46 | | |  |  | 31.66 | |  |  | 258.20 | |  |  | | | 206.53 | |  |  |
| **Model Comparison** |  | | |  |  |  | |  |  |  | |  |  | | |  | |  |  |
| - 2*log (lh) | 42053.42 | | |  |  | 40404.17 | |  |  | 46258.27 | |  |  | | | 46256.78 | |  |  |
| Diff - 2*log (lh) | 155.780*** | | |  |  | 238.951*** | |  |  | 190.028*** | |  |  | | | 129.847*** | |  |  |
| Number of Parameters | 21 | | |  |  | 21 | |  |  | 21 | |  |  | | | 21 | |  |  |

**Table S5**

Multilevel estimates for models predicting rumination (food, weight), affect, and tension. SE=Standard error, Group=-1(HC) 1(AN), Day=Day within study, Time=Prompt within day, Neural regulation score=positive watch-positive distance of extracted parameter estimates of ventral striatum, *** =p<.001

Table S6

| Multilevel estimates after exclusion of binge-purge subtype (n=3) for models predicting rumination (food, weight), affect, and tension for effects of interest only. | | | | |
| --- | --- | --- | --- | --- |
|  | **Food** | **Weight** | **Affect** | **Tension** |
|  | **p** | **p** | **p** | **p** |
| Neural Regulation | 0.03 | 0.015 | 0.587 | 0.805 |
| Neural Regulation x Group | 0.113 | 0.014 | 0.002 | 0.01 |

**Table S6:** p-values of estimated HLM Models for effects of main interest for the respective outcome after exclusion of three subjects with the binge-purge subtype.

Table S7

| Effect sizes of HLM Models | | | |  |
| --- | --- | --- | --- | --- |
|  | Level 2 |  | Level 3 | |
| **Food** |  |  |  | |
| **Complete Model** | 0.6 |  | 0.21 | |
| **VS×Group** |  |  | 0.02 | |
| **Weight** |  |  |  | |
| **Complete Model** | 0.54 |  | 0.46 | |
| **VS×Group** |  |  | 0.04 | |
| **Affect** |  |  |  | |
| **Complete Model** | 0.24 |  | 0.47 | |
| **VS×Group** |  |  | 0.08 | |
| **Tension** |  |  |  | |
| **Complete Model** | 0.26 |  | 0.1 | |
| **VS×Group** |  |  | 0.06 | |

**Table S7:** Delta-R² calculated to indicate explained variance at level 2 and 3

**Table S8**

| Multilevel estimates for models predicting rumination (food, weight), affect, and tension | | | | | | | | | | | | | | | |
| --- | --- | --- | --- | --- | --- | --- | --- | --- | --- | --- | --- | --- | --- | --- | --- |
|  | Food (a) | |  | | Weight (b) | |  | Affect (c) | | |  | Tension (d) | | | |
| Parameter | Beta | SE | | p | Beta | SE | p | Beta | SE | p | | | Beta | SE | p |
| **Fixed effects** |  |  | |  |  |  |  |  |  |  | | |  |  |  |
| Arousal Regulation | 0.76 | 1.77 | | n.s. | 1.44 | 2.25 | n.s. | 4.08 | 3.46 | n.s | | | 4.12 | 3.13 | n.s. |
| Arousal Regulation×Group | -1.62 | 1.77 | | n.s. | 2.25 | 2.24 | n.s. | -3.11 | 3.46 | n.s. | | | -3.89 | 3.13 | n.s |

**TableS8** Multilevel estimates for models predicting rumination (food, weight), affect, and tension. SE=Standard error, Group=-1(HC), 1(AN), Arousal regulation score=positive watch-positive distance of subjective arousal ratings

| Multilevel estimates for models predicting rumination (food, weight), affect, and tension | | | | | | | | | | | | | | | |
| --- | --- | --- | --- | --- | --- | --- | --- | --- | --- | --- | --- | --- | --- | --- | --- |
|  | Food (a) | |  | | Weight (b) | |  | Affect (c) | | |  | Tension (d) | | | |
| Parameter | Beta | SE | | p | Beta | SE | p | Beta | SE | p | | | Beta | SE | p |
| **Fixed effects** |  |  | |  |  |  |  |  |  |  | | |  |  |  |
| Neural Regulation | 3.15 | 2.28 | | n.s. | 2.94 | 2.21 | n.s. | -1.08 | 2.92 | n.s | | | -2.72 | 3.41 | n.s. |
| Neural Regulation×Group | 1.29 | 2.28 | | n.s. | 2.25 | 11.77 | n.s. | -5.54 | 2.92 | n.s. | | | -6.32 | 3.41 | n.s |

**Table S9**

**Table S9** Multilevel estimates for models predicting rumination (food, weight), affect, and tension. SE=Standard error, Group=-1(HC), 1(AN), Neural regulation score=positive watch-positive distance of extracted parameter estimates of right amygdala

**Figure S1**

**
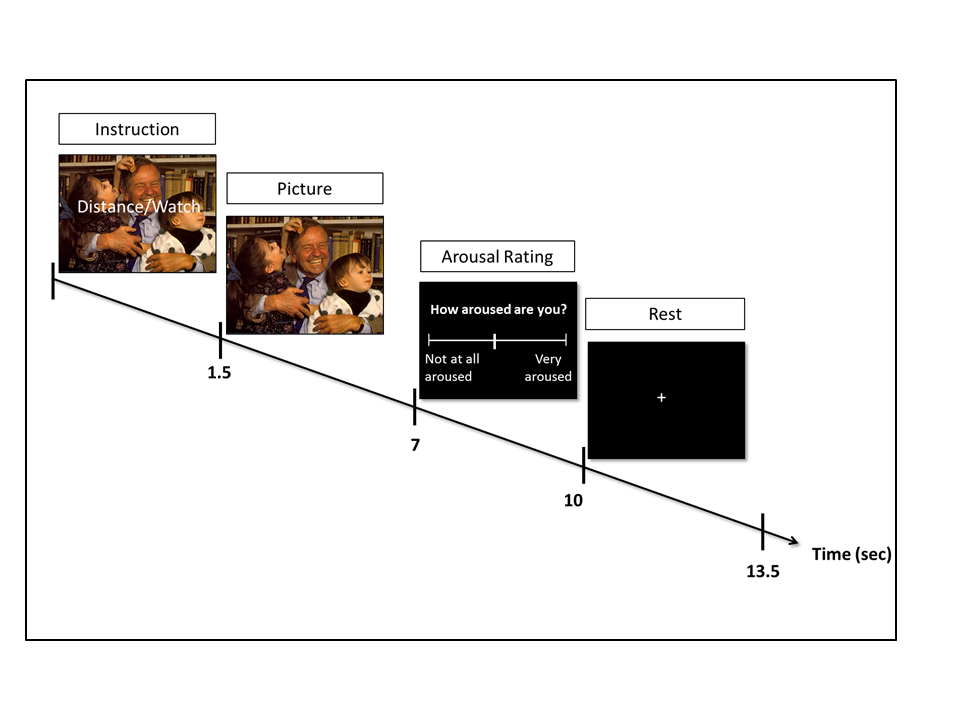
**

**Figure S1:** Setup of emotion regulation task. Stimulus was shown for 6.5 seconds with overlaid instruction (1.5s) “watch” or “distance”. Each stimulus was followed by the arousal rating (3s) and a jittered (average 3.5s) fixation cross. Time is shown in seconds.

**Figure S2**

**
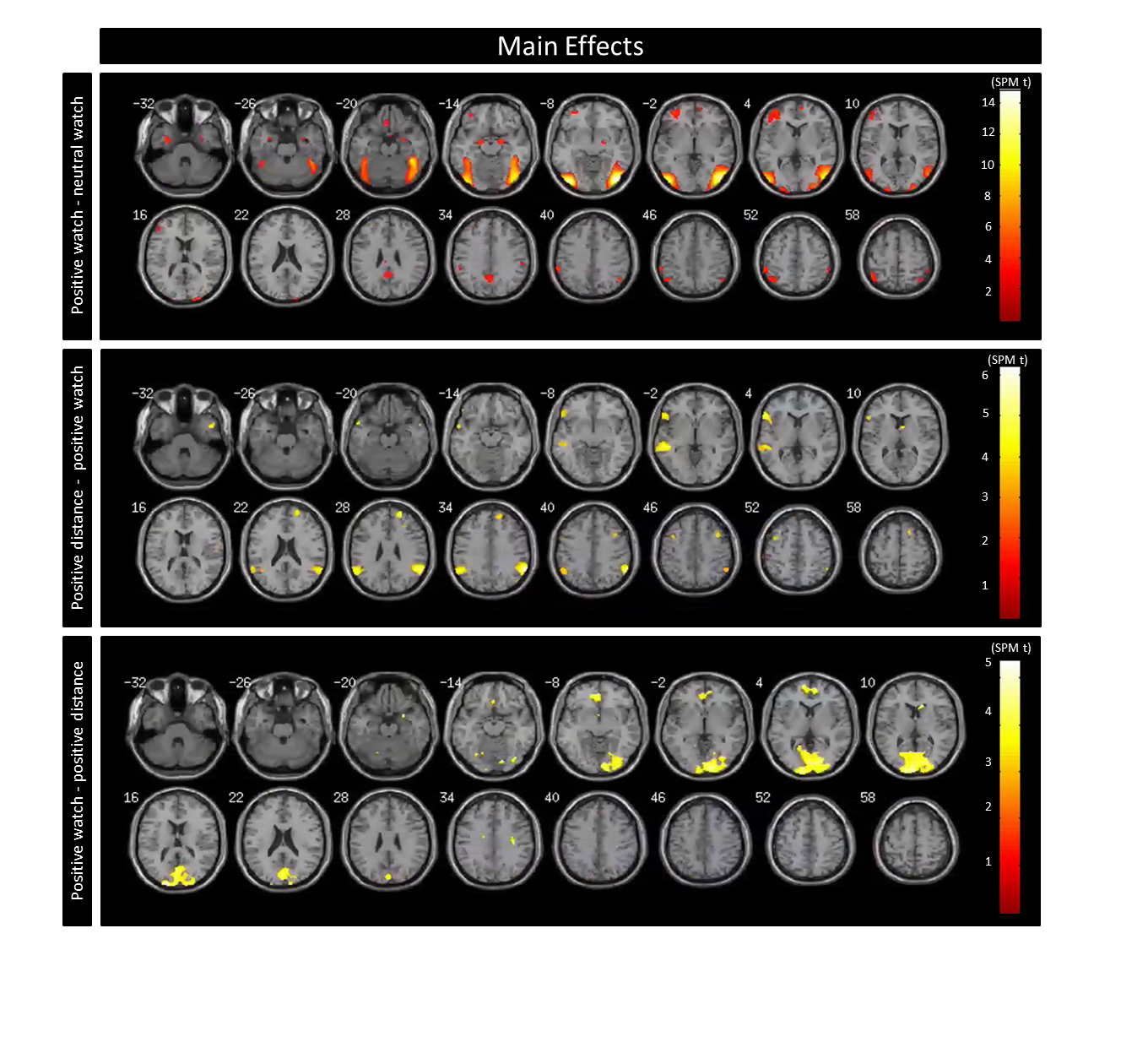
**

**Figure S2**: Whole brain main effects of emotion (posititive watch-neutral watch) and regulation (positive distance-positive watch, positive watch-positive distance). Brain maps are displayed at p<.001 (uncorrected) for display purposes only; Coordinates in Montreal Neurological Institute (MNI) space [x,y,z], labels represent z values; color bars represent t-values.

**Figure S3**

**
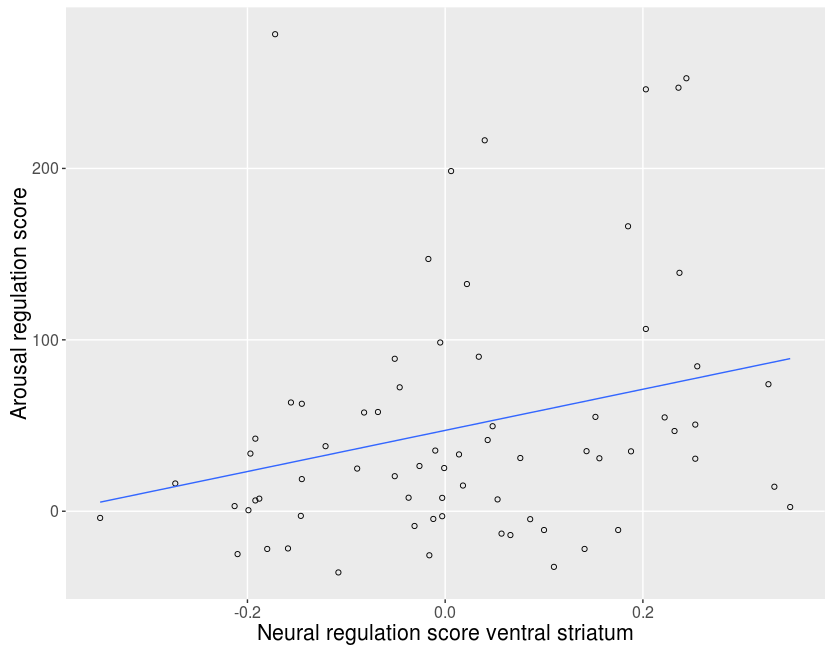
**

**Figure S3**: Correlation (including both groups) between the arousal regulation score (positive watch-positive distance of arousal rating) and the neural regulation score (positive watch-positive distance in the ventral striatum (rho=.27, p=.026)).

**Figure S4**

**
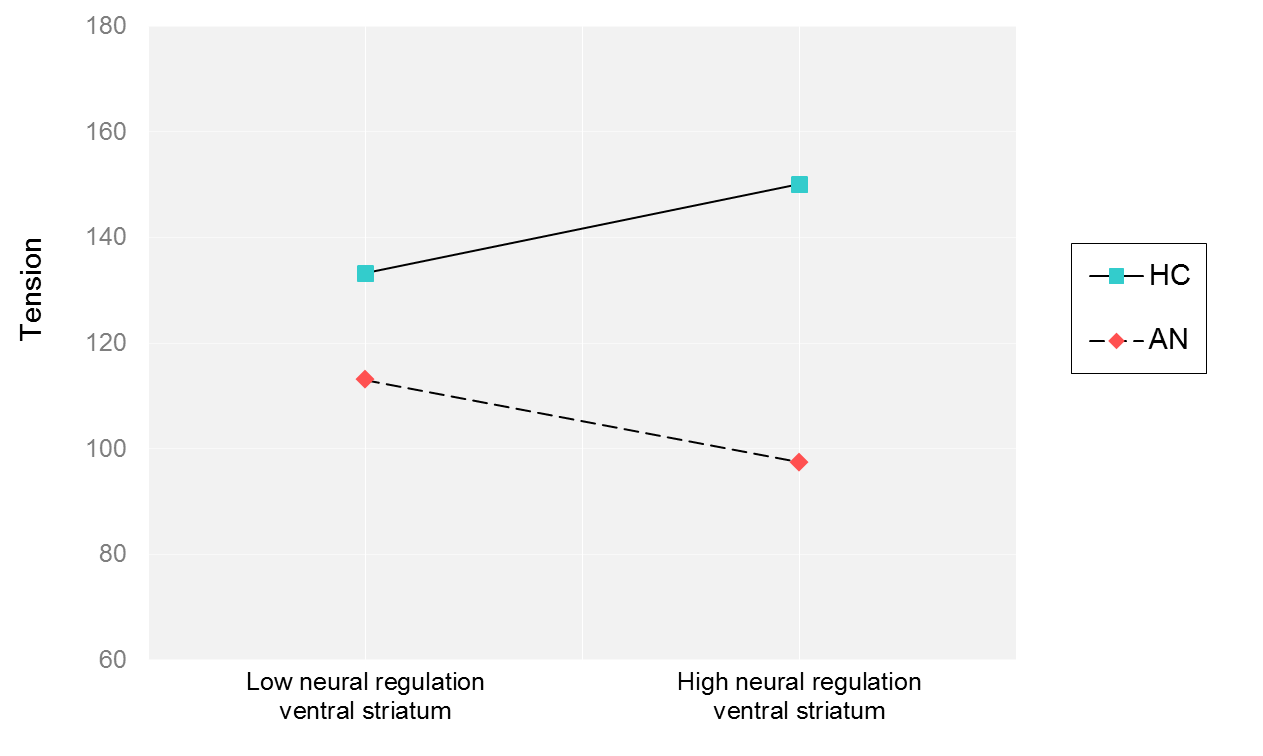
**

**Fig S4:** Neural regulation score x group interaction effect for tension as modelled by HLM. Simple slope analysis: HC: t=2.44, p=.015, AN: t=1.21, p=.23. AN = Anorexia nervosa; HC= Healthy control; VS= Ventral striatum. Low values indicate more tension. Neural regulation score of ventral striatum is dichotomous for display purposes only.

References:

1 Seidel M, Petermann J, Diestel S, Ritschel F, Boehm I, King JA *et al.* A naturalistic examination of negative affect and disorder-related rumination in anorexia nervosa. *Eur Child Adolesc Psychiatry* 2016; **25**: 1207–1216.

2 Fichter M, Quadflieg N. The structured interview for anorexic and bulimic disorders for DSM-IV and ICD-10 (SIAB-EX): reliability and validity. *Eur Psychiatry J Assoc Eur Psychiatr* 2001; **16**: 38–48.

3 Maas CJ, Hox JJ. Sufficient sample sizes for multilevel modeling. *Methodology* 2005; **1**: 86–92.

4 Petermann F, Petermann U. HAWIK-IV. *Kindh Entwickl* 2008; **17**: 71–75.

5 Von Aster M. *Wechsler Intelligenztest für Erwachsene: WIE; Übersetzung und Adaption der WAIS-III*. Harcourt Test Services, 2006.

6 Paul T, Thiel A. *Eating Disorder Inventory-2 (EDI-2): deutsche Version*. Hogrefe, 2005.

7 Hautzinger M, Keller F, Kühner C. Beck Depressions Inventar: Revised. 2009; **Huber: Bern**.

8 Ashburner J. A fast diffeomorphic image registration algorithm. *NeuroImage* 2007; **38**: 95–113.
